# Modelling Dunes from Lençóis Maranhenses National Park (Brazil): Largest dune field in South America

**DOI:** 10.1038/s41598-019-43735-0

**Published:** 2019-05-15

**Authors:** André Luís Silva dos Santos, Hélder Pereira Borges, Celso Henrique Leite Silva Junior, Raimundo Nonato Piedade Junior, Denilson da Silva Bezerra

**Affiliations:** 1 Federal Institute of Maranhão (IFMA), São Luís, Brazil; 2 National Institute for Space Research (INPE), São José dos Campos, Brazil; 3 Ceuma University (Uniceuma), São Luís, Brazil; 4 Present Address: Federal University of Maranhão - UFMA, São Luís, Brazil

**Keywords:** Environmental sciences, Environmental impact

## Abstract

This paper presents a digital elevation model (DEM) of the dunes found in the Lençóis Maranhenses National Park, an environmental protection area located in the Maranhão state (Brazil). The DEM supports the modeling studies of sand-dune evolution using multi-temporal satellite images and ground truth data, obtained through the post-processed kinematic Global Navigation Satellite System (GNSS) positioning. The study area is located at the border of three major neotropical ecosystems: the Amazonia, Caatinga, and Brazilian savanna. It is located in the northeastern state of Maranhão and encompasses the largest dune fields in the country. Wide shrubby areas (restingas, in Portuguese), lakes, mangroves, and a multitude off reshwater lagoons compose the park’s natural environments. The objective of the present study is to create an DEM that can evidence the complex dynamics of dune formation in the study area with use of GNSS. Geodetic techniques and precision mapping were employed to monitor the short-term coastal dynamics. The use of GNSS receivers is justified by the difficulty of mapping the dune’s features using conventional methods such as theodolite, level, and total station systems, due to their high cost, time restriction sand low data precision. Surface surveys were carried out annually between December 2015 and January 2017 to create a DEM. The study results reveal that the area has a negative volumetric balance of erosion and a preferential direction of sediment transport by wind, which may justify the pattern of advancement and retraction observed in the dunes of the studied area.

## Introduction

Coastal zone corresponds to the transition between the continental and marine domains, being subject to continuous morphodynamic changes that originate from the continental, marine and/or anthropogenic processes^1^. These processes are critical for the formation of different coast types, and include sea level oscillations, erosion, and depositional dynamics, associated with the action of waves, tides, marine currents and winds^2–4^. Being a highly unstable environment, the transition zone presents great temporal and spatial variability.

The Brazilian coast has a complex set of climatic, oceanographic and geomorphological elements that influence the active processes in certain areas, and subdivide the coast into distinct zones, such as the Amazonian Littoral, Northeastern Littoral, Eastern Littoral, and Southeastern Littoral^5,6^. The presence of dunes in the Northeastern Littoral zone is remarkable. Dunes are characterized as deposits formed by the action of wind on sand with or without vegetation^7^. Coastal dunes are notable for their role in protecting sea-level rise and are deemed important for conservation due to their vulnerability to natural and anthropogenic disturbances^8^.

Digital elevation models (DEM’s) are crucial in coastal dynamics studies providing a 3-D topographic model of the terrain^9–11^. The DEM’s can be used in: (1) the coastal evolution studies by feeding the predictive models and identifyingthe risk areas^12^, (2) the sustainable land use studies, and (3) the areas of intense coastal dynamics under the influence of anthropic activity. The DEMs can also be utilized in the analyses of sea-level rise and large-scale climate phenomena (e.g., El Niño and La Niña)^13,14^.

Previous information exists on the continental dune front advance in the Lençóis Maranhenses region^15,16^, however, no dune migration studies have utilized the GNSS methods in the surface of the dunes. This research is devoted to the generation of a DEM for the Lençóis Maranhenses National Park dunes using high-precision geodesy, dune-formation modeling studies, and field truth based on the GNSS data collection. The objective of the present study is to create a DEM that can evidence the complex dynamics of dune formation (processes of advancement and retraction) in the study area with use of GNSS.

## Study Area

**Figure 1:**
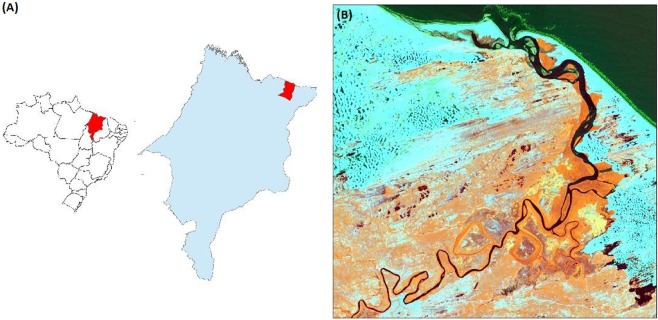
(**A**) Location of the study area within the continental Brazil, Maranhão state, municipality of Barreirinhas. (**B**) Satellite Image Landsat 8 OLI –Year of 2014 obtained at https://earthexplorer.usgs.gov/.

The study area is situated within the Lençóis Maranhenses National Park(NPLM), Maranhão, northeastern Brazil (central coordinates: 02°31′02′′S, 43°01′54′′W, WGS84). More precisely, a dune sector in the Village of Caburé (Barreirinhas municipality), near the Preguiças River mouth was studied (Fig. 1). This area is represented by transition from the Pre-Amazonian blackwoods to wide dune fields and mangroves. The NPLM park area constitutes approximately 155,000 hectares, and is composed of sand dunes, freshwater lagoons, restingas, lakes, mangroves and a 70 km beach strip. The dune field in the area has been interpreted to form as the result of: (1) retrogradation of sediments by construction of barriers in the Tertiary, (2) extension of the continental shelf, (3) successive marine transgressions since the Pleistocene, and (4) fluvial sediment input from the region’s main rivers^17^.The NPLM contains the Brazil’s largest dune field^18^ and was converted into a park in 1981. The NPLM represents chains of sinuous dunes interspersed with shallow, freshwater, temporary lagoons, making it the only “desert” with water on the Earth^19^.

Unlike other deserts, the NPLM receives relatively large amounts of water as precipitation (up to 2000 mm/year)^20^. The main form of precipitation is rain (>90%), falling between January and July. The rain is absorbed quickly by sand, and causes the rise of water table, thus forming the temporary lagoons (~1 m deep) between the dunes. High humidity, lack of wind, and elevated water table prevent the dunes from moving during the rainy season. The dunes gradually dry out and start moving in the second semester, when the prevailing winds blow from the east at 70 km/h.

The area studied in this work was a dune sector in Caburé village, near the mouth of the Preguiças River, Barreirinhas municipality, (Fig. 1). It is located in the Lençóis Maranhenses region, between 2°42′21′′ and 2°35′09′′ west longitude and between 42°470′15′′ and 42°39′56′′ south latitude. This region represents a transition between the Pre-Amazonia, Brazilian backwoods, and wide dune fields interspersed with mangroves, which are restricted to the river mouth and sand bays.

### Data acquisition and methodology

The location for data collection was chosen based on the satellite imagesof different scales. Two sensor image datasets (LANDSAT 5-TM (1986) and LANDSAT 8-OLI (2014)), both freely available from the US Geological Survey (website: https://www.usgs.gov/), were employed. The dune fieldsurvey was performed on different dates aiming to select the most appropriate location for the geodetic survey. Area accessibility and proximity to the region with evident shoreline changes were also taken into account.The images were georeferenced and RGB color-coded using the software ENVI 5®. The target area (near the villageof Caburé and the Preguiças River mouth) was identified usingthe software ArcGIS®, based on the most profound changes in the coastline architecture.

The geodetic survey was performed by satellite (GNSS) positioning and altimetry. DEM generation was based on the methodology proposed by another study^10,21^, that was later tested and validated by a number of other researchers^22–24^. The applied methodology consists of three phases: *data acquisition*, *data processing*, and *DEM creation*.

**Figure 2:**
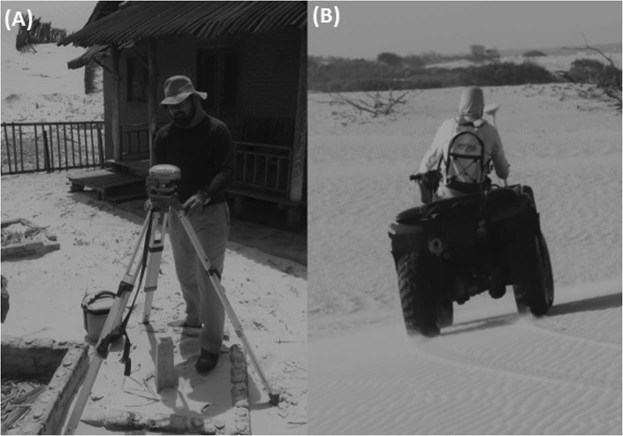
(**A**) Positioning of the geodetic mark No. RGLMA-01 in the NPLM dune field. (**B**) Collection of the GNSS dune survey data using a quadricycle.

The data acquisition stage was subdivided into: (1) positioning of the geodetic mark, and (2) planialtimetric survey of the dunes.The geodetic mark is a key survey point on the Earth’s surfacethat is necessary to carry out the planialtimetric survey by obtaining short vectors in the processing. The implementation of the geodetic mark (No. RGLMA-01, Fig. 2) followed the methodology outlined in another search^10^, and was based on the cadastral and geodetic marks specifications of the Brazilian Institute of Geography and Statistics (IBGE)^25^.

Coordinate navigation was carried out by the Geodetic L1,L2 Topcon GPS device (model Hiper+). The geodetic coordinates, ellipsoidal elevation and standard deviations were obtained using the Topcon Tools software to obtain the, according to the post-processing report, with IBGE’s RBMC stations, using São Luís and Teresina (Fig. 1). The static positioning method is the traditional GNSS measurement technique, consisting of data collection for a long session, between 1 and 8 hours. This method is ideal for long bases, with distances greater than 20 km, and it is used for implantation, control, densification of geodetic networks and several other precision works^26^. The geodetic planialtimetric survey of the dune surfaces was accomplished by the post-processed kinematic positioning (PPK) of the GNSS. The mobile receiver Trimble R3 with a horizontal accuracy of 10 mm + 1 ppm, vertical accuracy of 20 mm + 1 ppm and a 1 sec recording rate was set in a motorized quadricycle permitting faster data acquisition. The geodetic mark RGLMA-01 was used as a reference station, from which short bases to the GNSS positioning were employed, thus obtaining the geodetic coordinates with high precision.

The data processing phase consisted of: (1) determination of the geodetic coordinates, and (2) determination of the orthometric altitudes. The geodetic coordinates (i.e., latitude, longitude and geometric altitude) and the standard errors of captured points in the field were secured bythe GNSS data processing and adjustments. Data processing was carried out by the software Topcon Tools 7.5.1 with a fixed-type solution (fixed ambiguities), confidence level of 68%, and the standard permissible error in the vectors of 10 cm. The orthometric heights were calculated from the geometric altitudes of the revolution ellipsoid^26^. The geoidal model was obtained using the geoid undulation (N), where the orthometric altitude (H) was calculated by subtraction of the geoidal undulation (N) from the geodetic altitude (h), according to the equation (I): H = h − N. The interpolation by the triangulated irregular network (TIN) method was determined to be the most adequate technique for the representation of the sandy beach surfaces^27^. Thus, the DEM was generated using the TIN technique with the Delaunay triangulation in the software ArcGIS 9.3. Accordingly, the isolines were drawn from the original data layout with no extrapolation, and the estimates were limited to the area resulting from the sum of the areas of the triangles^21,28,29^.

## Results and Discussion

**Figure 3:**
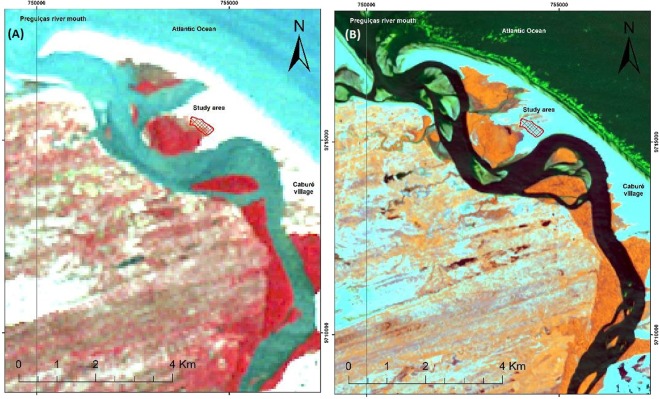
(**A**) Landsat 5TM –Year of 1986 obtained at https://earthexplorer.usgs.gov/. (**B**) Landsat 8 OLI –Year of 2014 obtained in site https://earthexplorer.usgs.gov/.

In Fig. 3, we can observe the changes which occurred in images acquired at an interval of 28 years: an increase of mangrove areas, dune and sand migration in a NE-SW direction (Fig. 3B), and a decrease in beaches of the region covered between Vila do Caburé and the study area.

Figure 3(A,B) presents satellite images from 1986 and 2014, with emphasis for the study area. Figure 3A presents an image of the Landsat 5TM series for the year 1986 with the color composition RGB_321, highlighting mangroves in red, beach and dune fields in white. Figure 3B shows an image of the Landsat 8 OLI satellite for 2014, showing a colored composition RGB_NDVI_PC1_B7 (NDVI, Main component 1 and Band 7), highlighting mangroves in orange, and beach and sand dunes in light cyan. The two images are processed in differently to reveal the main objects of interest: mangroves, sea, rivers, sand, among others.

**Figure 4:**
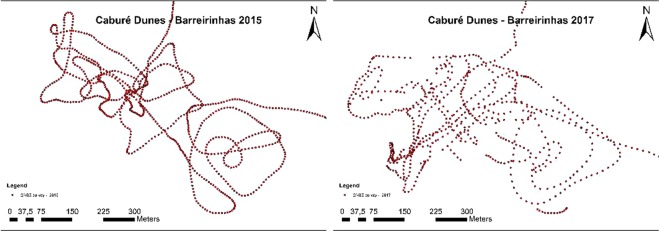
Point cloud of the geodetic survey conducted in the dunes of Caburé village, in January 2015 and January 2017.

Figure 4 shows the cloud of points obtained in the geodetic survey conducted in the dunes in December 2015 and January 2017. Respectively, a total of 1,010 and 1,700 sampling points with irregular distribution were collected, diffused according to the different morphological features found at the time of the survey. The precision obtained in GNSS data processing had an average of 1.0 cm in North, 2.4 cm in East and 2.5 cm in height for the UTM (Universal Transverse Mercator) coordinates.

Figure 3 shows points obtained at the base, top, and regions between dunes to accurately portray the morphology of the study region. The point cloud shows dune migration in the NE-SW direction illustrated in Fig. 4B. In 2015, it was not possible to access this region using a 4-wheeler.

**Figure 5:**
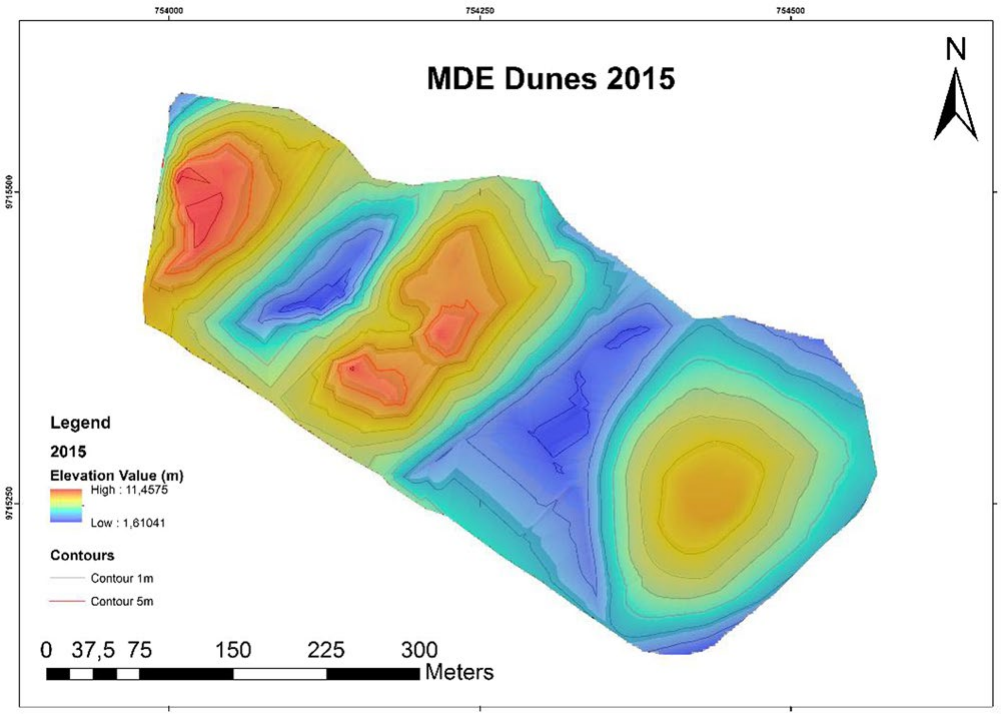
Digital elevation model of a dune monitored in 2015.

**Figure 6:**
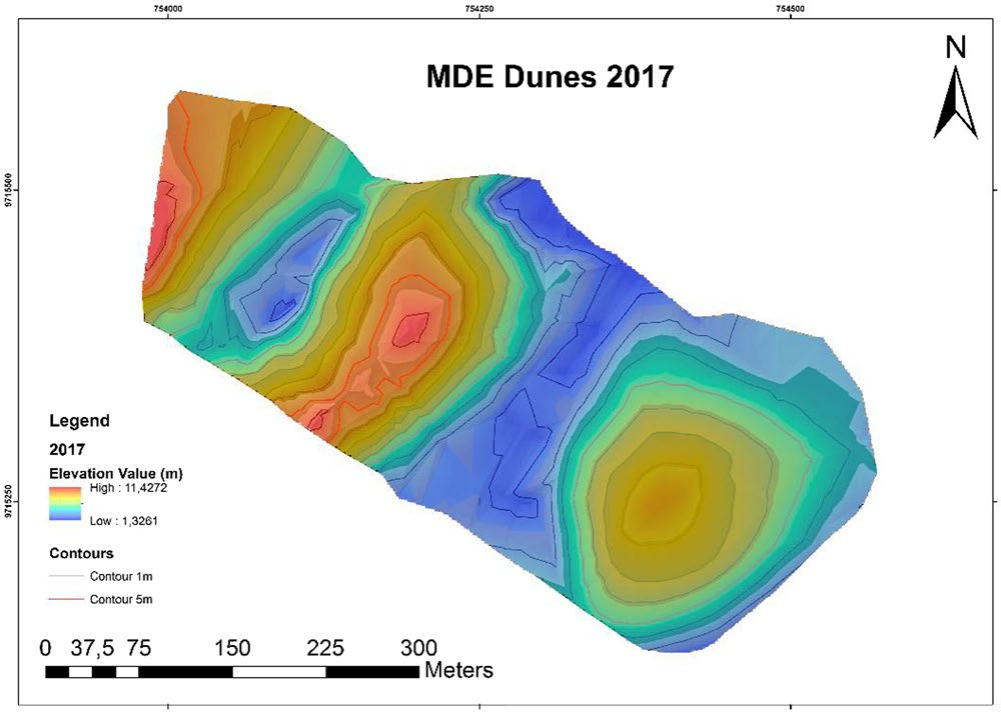
Digital elevation model of a dune monitored in 2017.

Figure 5 shows the DEM of 2015 and Fig. 6 presents the DEM of 2017. Both DEMs were obtained from the study area and by the geodetic method. The projection system used the UTM plan (Fuse 23 S) and the level curves generated had vertical intervals of 0.20 m, which was compatible with DEM accuracy. To improve the visualization of the models in the adopted scales, a color table with variations of 0.50 m was applied from blue (lower region) to red (highest region). The DEM has an altimetric precision of 2.5 cm and 0.50-cm resolution.

It was observed in the DEM of 2015 that the altitudes have an amplitude of 10.1471 m, ranging from 11.4575 m in the higher regions to 1.6104 m in the lower regions. In the 2017 DEM elevations, an amplitude of 10.1011 m was observed, varying from 11.4272 m in the highest regions to 1.3261 m in the lowest regions. In both models, the lowest regions was found between the dunes.

We observed that no relevant differences between low and high altitudes, or with range. However, visually, the DEM reveals dune migration we observed that there is no relevant difference in low and high altitudes (NE-SW). The direction of the migration is an important factor in the modeling process. The dunes are modeler by the wind, that’s the main actor to do it, like show^30,31^.

The relief of the area is typical of a dune region, with most altitudes concentrated between 1.0 m and 3.0 m (i.e., amplitudes of 2.0 m), accounting for 76.9% of the altitudes values between 1.0 m and 3.0 m, being 2.3% less than 1.0 m and 22.2% greater than 3.0 m). In the analyzed section, a pattern was observed in relief distribution, varying mainly according to location south of Caburé beach. The peaks present the highest altitudes, while the interdunal regions have the lowest altitudes. The model allowed the identification of morphological features typical of dunes in beach areas, such as the flat, horizontal and sloping profiles of the emerged beach face, small dunes in formation, depressions and elevations, as well as morphological features indicative of erosion^32^.

**Figure 7:**
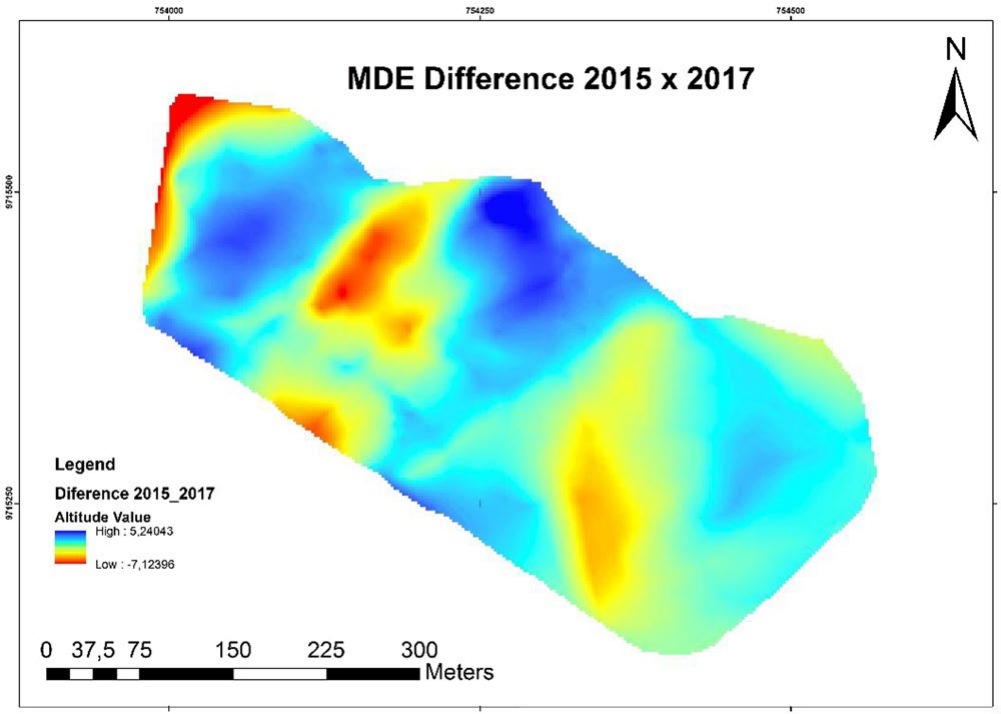
Volumetric variation between the DEMs of a dune monitoredin November 2015 and January 2017. Red areas indicate dune erosion and blue areas indicate sediment deposition.

Figure 7 shows the volumetric variation in the dunes of the study area for an annual monitoring interval between December 2015 and January 2017. Negative values in red represent altimetric loss (erosion) and positive values in blue represent altimetric gains (accretion). Thus, during the monitoring period, there was a decrease in sediment volume in the region with sediment displacement in the northeast to southwest direction.

Table 1 shows survey data, which is organized by a number of cloud points per year, minimum and maximum dune heights, and a total dune volume for each year.

**Table 1:** Survey Parameters.

Surveyyears	Point clouds	Minimumdune height(m)	Maximumdune height(m)	Dune volume (m^3^)
2015	1.010	1.6104	11.7575	838.018,78
2017	1.700	1.3261	11.4272	776.588,07
Difference	690	−0.2843	−0.3303	−61.43071

Using the software Area and Volume tool, 3D Analyst, a total volume of 838.018,78 m³ ± 2.5 cm was obtained for 2015, and a total volume of 776,588.07 m³ ± 2,5 cm was obtained for 2017. In other words, there was erosion on the order of 61.43071 m³ ± 2.5 cm in 1 year and 3 months of monitoring (Table 1). Figure 7 shows that the main areas of erosion are located at the top and bottom of the dunes and that sediment accumulation occurs to the southwest. Figures 4 and 5 indicate a decrease in the maximum altitude of the region, going from 11.7575 m to 11.42725 m, (i.e., a decrease of 33.03 cm). This is mainly seen in the red regions. The decrease in minimum latitude was similar to that occurring at the maximum altitude, being 28.43 cm.

## Conclusions

This research presents a multitemporal data analysis aimed for detection of the morphological changes in the dunes, located near by the village of Caburé, Lençóis Maranhenses region. The dunes have been deemed to experience the natural and anthropological changes (e.g., from the hotel construction businesses and tourist activities). This study uses a number of geodetic techniques and precision mapping to provide solutions for the short-term coastal dynamic’s problems. DEM supports the modeling studies of sand-dune evolution using multi-temporal satellite images and ground truth data, obtained through the post-processed kinematic Global Navigation Satellite System (GNSS) positioning. Several GNSS surface surveys were carried out annually between December 2015 and January 2017. The information has been collected and analyze dusing the digital elevation model (DEM). The study identified annual variations in the dune heights, recognized areas of sediment erosion and deposition, and allowed to understand the orientation of sediment particle transport and its balance. We have identified that the main dune change agent is the wind that carries the loose sand grains. The study results show that the area has a negative on-balance volume of −61.43071 m³, representing active erosion. More analyses are needed in the years to come in order to better understand the sediment dynamics and dune evolution in this transitional area of the Brazilian coast. Another perspective observed was the extreme relevance of the monitoring time extension of the morphological modifications of the region, indicating the necessity to repeat procedure in the following years. With more information, it will be possible to improve the understanding of the dynamics of sediment migration and dune evolution in the study area.
