# Author Correction: Modelling Dunes from Lençóis Maranhenses National Park (Brazil): Largest dune field in South America

**DOI:** 10.1038/s41598-019-54422-5

**Published:** 2019-11-28

**Authors:** André Luís Silva dos Santos, Hélder Pereira Borges, Celso Henrique Leite Silva Junior, Raimundo Nonato Piedade Junior, Denilson da Silva Bezerra

**Affiliations:** 1 Federal Institute of Maranhão (IFMA), São Luís, Brazil; 2 National Institute for Space Research (INPE), São José dos Campos, Brazil; 3 Ceuma University (Uniceuma), São Luís, Brazil; 4 Present Address: Federal University of Maranhão -UFMA, São Luís, Brazil

Correction to: *Scientific Reports* 10.1038/s41598-019-43735-0, published online 15 May 2019

The original version of this Article contained errors.

There was a typographical error in the spelling of the author Celso Henrique Leite Silva Junior, which was incorrectly given as Celso Henrique Leite Silva Júnior.

There was also an error in Affiliation 2, which was incorrectly given as ‘National Institute of Space Research (INPE), São José dos Campos, Brazil’. The correct affiliation is listed below:

National Institute for Space Research (INPE), São José dos Campos, Brazil

Additionally, the Acknowledgements section in this Article was incomplete.

“The authors are grateful for financial aid provided by the Maranhão Scientific Research and Technological Development Support Foundation (FAPEMA). The financial aid was received in the form of the Universal Edict and student scholarship grants. We also thank the Maranhão Federal Institute of Education, Science and Technology (IFMA) for providing the financial incentive through the Special Program for Internationalization of Research. The Geoprocessing Laboratory of the Federal University of Rio Grande do Norte is thanked for the geodetic GPS loan. The authors also thank Fapema the Coordination of Improvement of Higher Level Personnel - Brasil (CAPES) - Finance Code 001.”

now reads:

“We would like to thank the Fundação de Amparo à Pesquisa e Desenvolvimento do Estado do Maranhão - FAPEMA (Grant UNIVERSAL-00714/15), and the Universidade CEUMA - UNICEUMA for financial aid provided. This study was financed in part by the Coordenação de Aperfeiçoamento de Pessoal de Nível Superior - Brasil (CAPES) - Finance Code 001. Finally, we would also like to thank the Instituto Federal de Educação, Ciência e Tecnologia do Maranhão - IFMA for providing the financial incentive through the Special Program for Internationalization of Research, and the Laboratório de Geoprocessamento of the Universidade Federal do Rio Grande do Norte - UFRN for the geodetic GPS loan.”

These errors have now been corrected in the PDF and HTML versions of the Article.

